# Optimizing wheat development to a range of winter climates

**DOI:** 10.1093/jxb/erag129

**Published:** 2026-03-16

**Authors:** Dominique Hirsz, Laura E Dixon

**Affiliations:** Genebank Department, Leibniz Institute of Plant Genetics and Crop Plant Research (IPK), Gatersleben, Seeland D-06466, Germany; Genebank Department, Leibniz Institute of Plant Genetics and Crop Plant Research (IPK), Gatersleben, Seeland D-06466, Germany; Università degli Studi di Modena e Reggio Emilia, Italy

**Keywords:** Adaptation, changing climate, vernalization, winter wheat

## Abstract

The effects of climate change are highly disruptive for reliable and sustainable crop production as crops have been regionally adapted to respond favourably to a set of regular, combined environmental cues. Notably in wheat, the most widely cultivated crop, the timing of floral meristem transitions and flowering is largely regulated by the combination of photoperiod and temperature cues. Identifying and understanding the key genes that regulate the physiological responses to these combined environmental cues has been important for enabling the optimal development of cultivars. Winter-grown crops are important as they provide ground cover, high biomass, and high yield potential. However, they are critically sensitive to the duration and level of cold season temperatures and the onset of the lengthening spring photoperiod. Therefore, to enable climate-robust cultivars, we need to understand and tailor the crop response to the winter environment; the crop must be resilient enough to survive but flexible enough not to require a standard winter each year. Here we detail the challenges and opportunities that are presented by the changing environmental conditions for the adaptation of winter wheat.

## Teaser

Through characterizing and understanding genetic pathways that regulate floral development, we can move away from classing winter wheat as vernalizing or not and towards a tailored winter growth habit.

## Introduction

Weather patterns are major regulators of crop yield, but it is notoriously hard to identify specific trigger conditions for which we can develop resilient germplasm. This is largely due to the complexity of predicted climate change patterns, which are variable across the world, mainly caused by faster warming on land masses compared with the oceans and variation in wind patterns (Intergovernmental Panel on Climate Change, 2021). Warming is predicted to alter the northern hemisphere disproportionately to the south, increasing closer to the Arctic due to the phenomenon of ‘Arctic amplification’ (Rantanen *et al*., 2022). Europe is the continent that is warming most rapidly and is expected to experience an increase in extreme weather events and heatwave days (European Environment Agency, 2024). However, other effects are variable depending on region; in northern Europe a higher level of rainfall and associated flooding risk is anticipated, whilst in central and eastern Europe an increase in drought events is predicted (Trnka *et al*., 2015; Webber *et al*., 2018; European Environment Agency, 2024). In the UK, climate predictions indicate favourable conditions for wheat growth in the north resulting in higher yields, whilst in the south of the UK yields are predicted to decrease (Hayman *et al*., 2024). Yield decreases are predicted for the Australian wheatbelt by the end of the 21st century, as there will be less suitable land for growing wheat (Wang *et al*., 2018). By contrast, the North China Plain, which produces over 50% of the country’s grain, is predicted to have an increase in yield with an increase of up to 2 °C, partially due to increased rainfall (Hu *et al*., 2025; Wang *et al*., 2025). Much like the variation in temperature, changes in rainfall patterns are predicted to have both positive and negative impacts with an increase in drought events in many spring wheat-producing parts of the world. Further, current climate modelling approaches may underestimate the severity of extreme weather events including flooding, indicating the challenges may be greater than currently predicted (Noia *et al*., 2025). Many models do not consider other issues such as the spread of diseases, with the expansion of wheat blast predicted to cause a major decrease in yield in South America (Pequeno *et al*., 2024). The increase in unpredictable weather conditions and extreme events will complicate future breeding efforts, as making selections will become less consistent year-to-year under inconsistent environmental conditions.

In the context of the changing climate, developing cultivation patterns for robust yields becomes more challenging. Whilst there are many benefits to growing winter wheat, the requirement for low ambient temperatures to enable the vegetative to floral transition means that it is vulnerable to increasingly variable autumn and winter temperatures (Antoniou-Kourounioti *et al*., 2021; Fradgley *et al*., 2023). It also leaves the plants extremely vulnerable to the optimal alignment of temperature and photoperiod signals (Zhang *et al*., 2022; Fradgley *et al*., 2023).

Our current molecular understanding makes it hard to predict the impact of warmer temperatures on vernalization, the requirement for an extended period of low temperatures before the vegetative to reproductive transition can be made. Shifting temperature patterns leading to warmer autumns may delay or advance the onset and progression of vernalization depending on the cultivar, impacting the duration of plant growth and yield potential. Equally, altered vernalization patterns can lead to a reduced synchrony in the final flowering time that is important to maximize the duration of grain filling and the final grain number for harvest (Sheehan and Bentley, 2021). Increased variability during autumn and winter is anticipated to impact how vernalization proceeds due to the process of devernalization. Under variable conditions that include higher ambient temperatures, the vernalization process is stalled or perhaps reversed (Dixon *et al*., 2019).

Temperature during vernalization is an important variable for selection alongside duration. Usually, artificial vernalization is conducted at a constant day and night temperature of 4–6 °C for 8 weeks (Dixon *et al*., 2019). This can be lower than is experienced by many crops under field conditions (Gauley and Boden, 2021). Furthermore, this temperature is when molecular processes slow down and the plant is preparing for cold stress (Hassan *et al*., 2021). In line with this, a study has shown that vernalization actually proceeds most efficiently between 8 and 12 °C (Yan and Hunt, 1999). However, this study only considered a few cultivars, so it is reasonable to expect that there will be cultivars that vernalize efficiently at temperatures below 8 °C. This is supported through the identification of recalcitrant lines that are believed to require lower temperatures to fully vernalize. A second possibility is that the recalcitrant lines vernalize in the same temperature range as other cultivars but require a lower post-vernalization temperature to ensure the vernalization response is established. Whilst this is unknown it would be a very interesting aspect to consider in wheat, as the main molecular regulator, *VERNALIZATION 1* (*VRN1*), is regulated partly via epigenetic marks controlling expression, which suggests this aspect could be fine-tuned through selection (Oliver *et al*., 2009; Huan *et al*., 2018). It also puts the focus on the temperatures of both winter and early spring. Both of these aspects need further consideration to develop wheat cultivars that have a predictable and robust response.

**Fig. 1.**
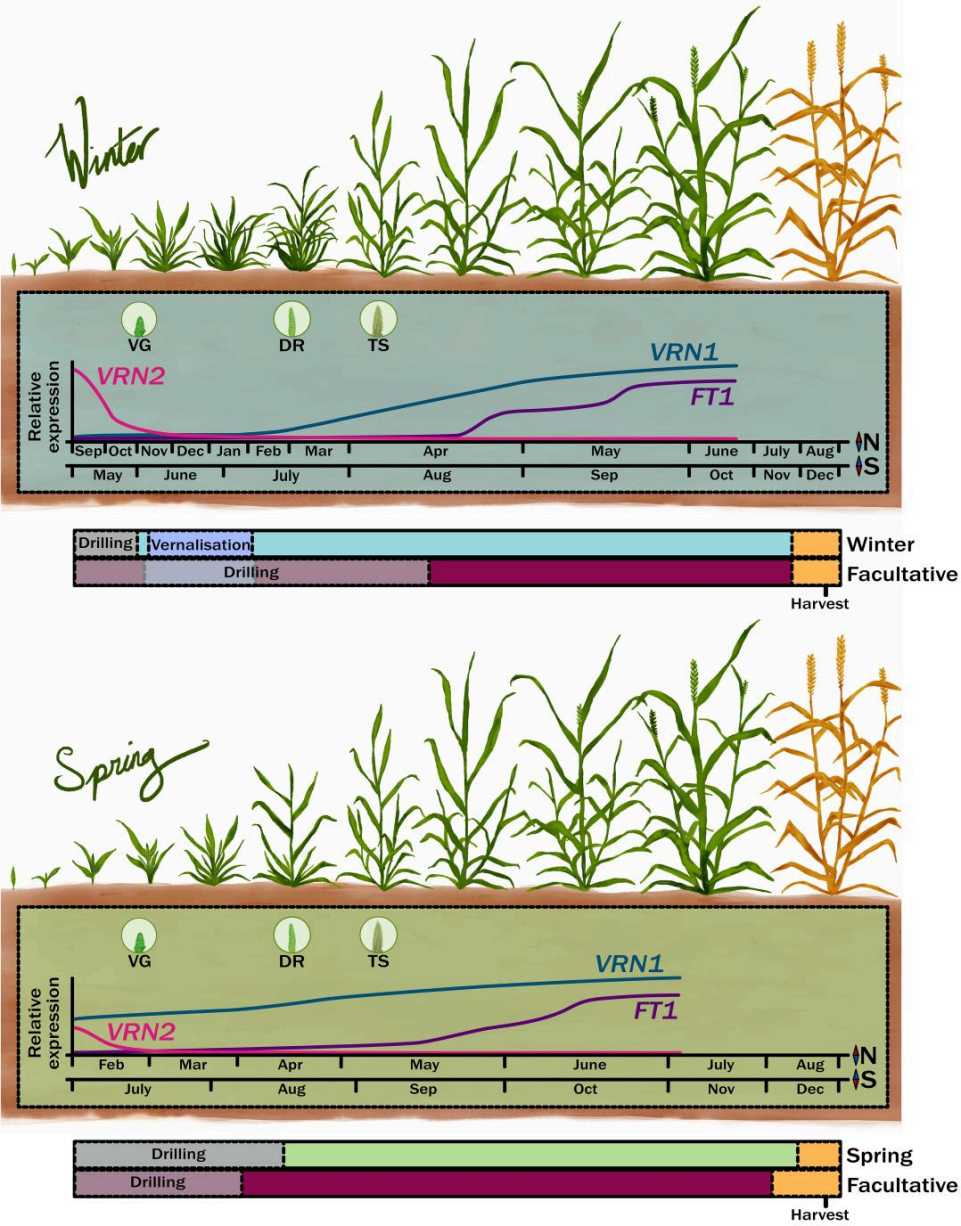
The major wheat growth habits can be tracked through changes in gene expression. A schematic representation of the progression of the two growth habit crops (winter and spring) over a typical high latitude growing season (N) and a low latitude growing season (S). The timing of apex development is indicated as VG (vegetative), DR (double ridge), and TS (terminal spikelet) and is based on a typical UK growing season representing high latitudes and Australian growing seaseon representing low latitudes. Three major genes are associated with winter and spring growth habit in wheat. *VERNALIZATION 1* (*VRN1*) (blue line) is a MADS-box domain floral promoter the expression of which increases to enable reproductive growth. *VERNALIZATION 2* (*VRN2*) (pink line) is a CCT and putative zinc-finger domain floral repressor, the expression of which decreases with shortening days. *FLOWERING LOCUS T1* (*FT1*) (purple line) is a PEBP domain floral promoter and integrator of floral promoting signals. The increase in expression of *FT1* follows vernalization under winter conditions. Beneath each growth habit the cultivation timeline is represented through drilling (grey bar), vernalization (dark blue bar), growth (blue, green, or magenta depending on cultivation programme), and maturity and harvest (orange bar).

Finally, an important consideration is regionality of winter stresses (Fig. 1). For example, frost damage is not experienced at the same time in all environments, and it remains unclear if the genetic factors that provide winter hardiness at the end of vernalization would still provide protection if the frost was experienced much later in the season in a spring background. Ascertaining this is important as in the Australian environment spring wheat can experience damaging frost events when the plant is undergoing stem elongation (GS31), booting (GS45) and even emergence (GS58) (Frederiks *et al*., 2015; Zheng *et al*., 2015). These post-head-emergence frost events are catastrophic, with losses in yield of up to 85% (Zheng *et al*., 2015).

## Seasonal adaptation of bread wheat

### Seasonal adaptation uses both temperature and photoperiod

Of the top three small grain cereals that underpin modern agriculture, only wheat, the allotetraploid *Triticum durum* and the allohexaploid *Triticum aestivum*, is widely grown across the winter season. Bread wheat (*Triticum aestivum*) is used for many food products, providing essential calories and protein to millions across the globe, and forming a high protein component of animal feed. Genetic and archaeological evidence identifies wheat as an origin plant in the establishment of agricultural communities, and this early origin and popularity has enabled the development of regional specialty cultivars (International Wheat Genome Sequencing Consortium, 2018; Levy and Feldman, 2022). Furthermore, part of its widespread cultivation success has been due to the apparent ease and speed with which the early wheat could adapt to new environments (Levy and Feldman, 2022).

We now categorize this early adaptation by two factors; growth habit (winter, spring, or facultative based on the plant’s vernalization response) and photoperiod sensitivity (sensitive or insensitive). The key mutations that enabled this adaptation happened spontaneously and have subsequently been selected for during cultivation (Cheng *et al*., 2024). This process is particularly interesting when considered from an evolutionary standpoint, where the origin grasses were probably short-day, spring habit (Verrico and Preston, 2025). In both cases of what we believe to be the change in the domesticated wheats of winter to spring habit and photoperiod sensitive to insensitive, the mutations have occurred in a single copy of a gene and enabled a dominant gain-of-function allele. For winter/spring habit this was through mutations in the promotor or intron in the gene *VERNALIZATION 1* (*VRN1*) on chromosome 5A, and for photoperiod responses it was mutations in *PHOTOPERIOD 1* (*Ppd-1*) on chromosome 2D (Trevaskis *et al*., 2003; Yan *et al*., 2003; Turner *et al*., 2013). The subsequent selection of these responses was essential to enable the crop to move across the globe from the origin of domestication in the Fertile Crescent (International Wheat Genome Sequencing Consortium, 2014). In subsequent genetic studies the identification of allelic series has enabled a start to the quantification of the contribution of each of the hexaploid wheat genomes to the response (Beales *et al*., 2007; Shaw *et al*., 2012, 2013; Bentley *et al*., 2013; Kiseleva *et al*., 2017; and references within Milec *et al*., 2023). This has provided valuable information on both growth habit and photoperiod sensitivity and has also identified the existence of copy number variations for both of these genes (Diaz *et al*., 2012; Kippes *et al*., 2014).

### Physiological and molecular control of winter responses

The major environmental signal that regulates growth habit is the requirement for vernalization before winter-type plants will proceed through the vegetative to reproductive transition (Fig. 1). This can be sub-categorized according to the degree and duration of low temperatures to enable reproductive development (Luo and He, 2020). Ensuring that the winter type is matched to the environment has been essential in maximizing potential grain yields (Sheehan and Bentley, 2021). The sub-categorization of the response has identified facultative growth habit wheat that can flower without experiencing vernalization, but if the plants are vernalized they flower faster (Fjellheim *et al*., 2014). However, there is another definition of facultative wheat that will become more important with climate change, and that is a wheat that can productively survive a winter and spring planting, irrespective of vernalization requirement. The emergence of such a wheat type highlights the need to consider vernalization and winter hardiness independently. Traditionally, the vernalization requirement is important for adaptation to enable a high biomass, ground cover, and yield potential. However, for true winter types the risk exists that the winter may be too warm to meet the minimal vernalization requirement. If this occurs, the crop will continue to grow as a vegetative grass, producing no or very low yield.

Vernalization is well studied in many plant species (Luo and He, 2020). It is rare in annual plants that the vernalization response is an absolute requirement, as normally plants will eventually flower. For example, the reproductive transition is triggered by age or other environmental or developmental cues. However, this usually affects only one meristem and so results in the development of one floral stem in a mass of leaves and represents a complete loss of agricultural yield as the plant flowers too late to harvest. Therefore, in the context of understanding and applying the vernalization response, a true winter type is generally considered to be a plant that requires a prolonged period of low temperatures (8–12 weeks) before transitioning to flowering.

Developmental and physiological characterization of the vernalization response identified that it is a quantitative response, up until the point of saturation. This is such that a plant with a vernalization requirement of 6 weeks, when it has experienced 4 weeks of cold would flower earlier than when it had only received 2 weeks of cold temperatures (Kim *et al*., 2009; Dixon *et al*., 2019). Additionally, the cold temperature that enables vernalization is species- and even cultivar-specific (Yan and Hunt, 1999; Kim *et al*., 2009; Duncan *et al*., 2015). The rate of vernalization has been linked to a few molecular mechanisms including single nucleotide polymorphisms (SNPs) in the RIP3 domain of *VRN-A1* (Kippes *et al*., 2018), as well as coding sequence polymorphisms (Li *et al*., 2013) and potentially copy number variation of *VRN-A1* (Diaz *et al*., 2012). The existence of mechanisms that enable the distinction of temperatures makes adaptive sense as plants growing in regions that experience colder winters need to be able to distinguish autumn and winter temperatures (Fig. 1).

**Fig. 2.**
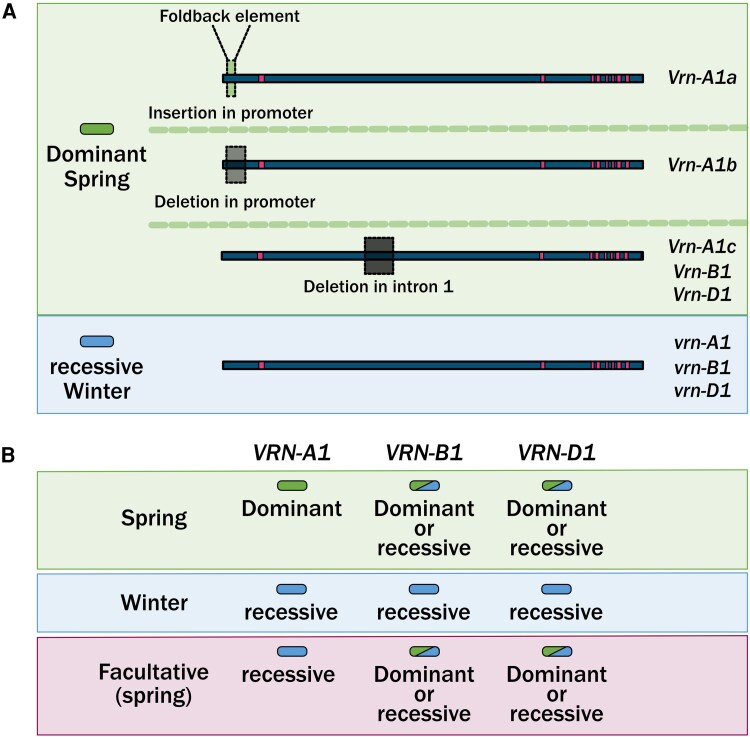
Types of growth habit regulation via *VRN1* allelic variations. Hexaploid wheat usually contains at least three versions of *VERNALIZATION* (*VRN1*) gene, one on each of the sub-genomes, all of which can be selected for independently to modify the growth habit response of wheat. Mutations that increase the expression of either *VRN-A1*, or *-B1*, or *-D1* are dominant or semi-dominant and are represented here by green rectangles. Allelic variation that leads to changes in the vernalization duration and have been characterized in winter wheat is represented here as blue rectangles. (A) The major characterized alleles of *VRN1*. (B) The diverse combinations of dominant spring and recessive winter alleles that can form to generate the different types of growth habit.

At the molecular level a true winter wheat contains recessive *VRN1* alleles on all sub-genomes and in all genomic locations (Fig. 2), as *VRN1* has also been translocated from its original position on the Group 5 chromosomes (Kippes *et al*., 2014). Alleles that enable gain-of-function through increased *VRN1* expression result in a reduced or a loss of vernalization requirement depending on the sub-genome where the allele is situated. In addition to *VRN1*, a second genetic locus has been identified that is important for vernalization in cereals, *VERNALIZATION 2* (*VRN2*). *VRN2* is formed of two genes, *ZCCT1* and *ZCCT2* (where ZCCT refers to the domains of the protein; zinc-finger, CONSTANS, CONSTANS-like, and TOC), which originated via a gene duplication but are now only ∼80% similar (Yan *et al*., 2004). These genes contain a putative C2H2 zinc finger domain and a CCT domain, and gene expression is repressed under short-day photoperiods (Yan *et al*., 2004; Hirsz *et al*., 2026). These *VRN2* genes have undergone chromosomal translocations with the A-genome *VRN2* genes on chromosome 5A whilst the B and D-genome *VRN2* genes are on chromosomes 4B and 4D (Ma *et al*., 2013; Hirsz *et al*., 2026). In the diploid grasses, including barley (*Hordeum vulgare*) and *Triticum monococcum*, a deletion of the *VRN2* locus results in the spring habit (Yan *et al*., 2004; Dubcovsky *et al*., 2005). This suggests that these genes are as important as *VRN1* in regulating the vernalization response. However, in tetraploid and hexaploid wheat it is more complicated as deletion of the genes on any one of the genomes does not result in the spring habit (Chen and Dubcovsky, 2012; Kippes *et al*., 2016). Furthermore, for the vernalization response a functional CCT domain is required (Li *et al*., 2011). However, it remains the case that non-functional alleles at the CCT domain may have a role in other responses. The nature of the hexaploid genome therefore offers a further expansion of fine-tuning opportunities for the vernalization response (Hirsz *et al*., 2026). Through selecting these regions, a wide range of vernalization responses and important associated plant developmental traits could be achieved.

Beyond these two genetic regions there is a further target for growth habit adaptation, *VRN3* (Yan *et al*., 2006). The *VRN3* gene is an ortholog of *FLOWERING LOCUS T* (*FT*) in Arabidopsis and integrates floral promoting signals in both species. In hexaploid wheat the B-genome has been associated with the vernalization response for the *FT1* gene (*FT-B1*) (Yan *et al*., 2006). Allelic variation of *FT-B1* has confirmed its central role in floral regulation, with an allele that enables a naturally higher level of expression (*Hope-1*) flowering earlier (Nitcher *et al*., 2014). Additionally, deletions of *FT1* have been identified. These have mostly been characterized in spring backgrounds, and notably the plants still flower, although significantly delayed (Dixon *et al*., 2018; Finnegan *et al*., 2018). Therefore, it highlights that other *FT1* and possibly other *FT* genes are acting in addition to *FT-B1* to promote flowering (Bennett and Dixon, 2021). This raises the possibility of understanding these genes and their contribution to vernalization (Fig. 2). For example, a spring cultivar lacking *FT-B1* may act like a winter habit wheat regarding its rate of development but not actually require vernalization. Additionally, exploring the impacts of other *FT* genes may provide a less severe delay in flowering but significant early developmental delays as suggested from research in wheat and barley (Zikhali *et al*., 2017; Shaw *et al*., 2019; Gauley and Boden, 2021; Pieper *et al*., 2021; Gauley *et al*., 2025).

To robustly adapt winter wheat for variable climate conditions, the timing of development should be considered beyond that of the point of vernalization saturation. Here the role of photoperiod signals becomes increasingly important. Photoperiod alone could be an unreliable seasonal indicator as both autumn and spring have shorter daylengths. However, combined with temperature cues, or as a sequential changing duration measure, photoperiod is an important seasonal signal. This has been explored regarding photoperiod sensitivity and growth habit, such that the dual selection of these two loci via marker assisted selection (MAS) is now common. Expanding this to include minor allelic variants of the *PPD-1* and *VRN1* genes remains important. Photoperiod regulation has also been shown to contribute to the regulation of the vernalization response either directly through regulating the expression of genes or indirectly through circadian clock-associated regulation (Dubcovsky *et al*., 2006). Importantly in the seasonal timing of vernalization, *VRN2* is repressed by short-day photoperiods, and so as autumn progresses, the floral repression via *VRN2* is lifted (Dubcovsky *et al*., 2006). Additionally, and of considerable interest, is the close proximity and involvement of *PHYTOCHROME C* (*PHY-C*) to *VRN1* (Chen *et al*., 2014). The nature of the combined and interacting pathway is unresolved in wheat but the *PHY-C* mutants have very strong impacts on the timing of flowering. Furthermore, testing unusual combinations with frost sensitive genes or short-day florigens would also enable the development of wheat that has a predictable and robust response to the environment. Increasingly the *FT-like* genes of wheat are being shown to have a role in temperature and photoperiod responses. The short-day florigen *FT3* has also been linked with lower ambient temperature responses that, combined with allele selection from diversity collections, highlights an important role in the winter to spring transition (Gauley *et al*., 2025).

Interestingly, there are examples of plants, including *Brachypodium distachyon*, *Bromus inermus*, and *Phalaris arundinacea*, that are able to undergo a process of short-day vernalization (Purvis and Gregory, 1937; Heide, 1994; Woods *et al*., 2019). Here, rather than the low temperature signal, plants require a period of short days, similar to the low-temperature response, which enables a quantitate response to the signal and earlier flowering (Verrico and Preston, 2025). Without the short-day signals, the plants do not go on to flower (Purvis and Gregory, 1937; Heide, 1994; Woods *et al*., 2019). Mobilization of such a response to wheat could be highly impactful.

Importantly via either low temperature or short-day pathways the effects of vernalization are only associated with one generation, such that the seed produced from a vernalized plant have lost the seasonal memory of winter and can therefore be vernalized. This is important as seeds could germinate in any season, and so requiring winter prevents these annual plants from mistiming the reproductive transition (Kim *et al*., 2009).

### Fluidity of the seasonal response

A further temperature response that adds an additional challenge to adapting the vernalization response is the process of devernalization. Early reports of devernalization used very high temperatures of 34 °C (Gregory and Purvis, 1948) that, at the physiological level, remove the effect of the vernalization response. At the biological level this scenario is currently extremely unlikely. Very rarely, if ever, are plants going to experience the high temperatures needed for devernalization response during the winter period. Therefore, from a biological perspective, the high temperature devernalization response is most likely related to ensuring plants do not inadvertently use a cool spring as a vernalization trigger. Additionally, the high temperature devernalization response may be related to a now lost trait in the cultivated crops (but a common trait in non-cultivated grasses) of perennialism. Here devernalization of any meristems that had not grown through in the first seasonal cycle would provide the opportunity for these to become vernalized with the following winter, so facilitating the winter habit. This is important to consider from the perspective of possible multiple origins of vernalization occurring in the grasses, and therefore some of the responses that we now observed may actually relate to traits that are no longer present in that species (Verrico and Preston, 2025).

However, relating to vernalization there are reports of a different type of devernalization that occurs in field and controlled conditions (Verrico and Preston, 2025). Here, the vernalization requirement is extending due to periods of warmer temperatures, such as those experienced during a mild winter or on those occasional warmer days experienced even during a harsh winter. The lack of complete vernalization is anecdotally reported to cause a significant delay in flowering and therefore loss in final yield. This suggests that the process of vernalization in cereals is not occurring in a single direction and that variable winter temperatures can significantly impact the process (Bouche *et al*., 2015; Dixon *et al*., 2019; Kennedy *et al*., 2024). Such a response is reminiscent of the molecular characterization of vernalization that has been conducted in Arabidopsis. Here it has been observed that the establishment of epigenetic marks that enable the cumulative summation of the winter period is not fixed until the plant experiences spring-like temperatures (Hepworth *et al*., 2018). With this the epigenetic mark that enables the silencing of the floral repressor gene occurs and vernalization is fixed (Berry and Dean, 2015; Questa *et al*., 2020; Zhu *et al*., 2021). Additional further molecular and mathematical modelling characterization of the vernalization response has identified that there are many routes for temperature input to vernalization (Hepworth *et al*., 2018; Antoniou-Kourounioti *et al*., 2021). This strongly indicates that we need to understand the response in cereals under variable conditions, as how vernalization proceeds and responds to seasonal changes may be quite different from that in controlled growth chambers.

A final aspect of regulation that has been characterized in Arabidopsis vernalization response and that it may be possible to translate into cereals to enable further fine-tuning of the response is that of long non-coding RNA (lncRNA). In Arabidopsis a population of lncRNAs have been identified originating from the 5′ untranslated region (Swiezewski *et al*., 2009). This population is collectively described as *COOLAIR* and is involved in the regulation of *FLC* expression. Natural variation in the level of *COOLAIR* expression and epigenetic regulation of the FLC gene and its expression have been linked with the variation of vernalization requirement (Shindo *et al*., 2006; Hawkes *et al*., 2016; Hepworth *et al*., 2020). Furthermore, lncRNA related to *VRN1* in wheat has also been shown to influence flowering regulation (Xu *et al*., 2021).

## Adaption characteristics of winter wheat

### Winter wheat and cold hardiness

Winter hardiness comprises tolerance to many abiotic and biotic stressors such as frost, ice encasement, snow mould, and winter drought. Winter wheat generally has a greater cold tolerance than spring wheat, but once germinated it is not able to survive sustained, extreme low temperatures (Fowler, 2012). Therefore, winter wheat is not generally grown in areas that experience very harsh, long winters such as in regions of Canada and Asia. However, the practice of sowing seeds into the previous harvest stubble has enabled improved winter survival and the expansion of the wheat cultivation area (Fowler, 2012). More recently, and anecdotally, there are examples of sowing just before snow and that the seeds remain dormant over winter and are able to germinate as soon as the snow melts, taking full advantage of early spring moisture. This is not usually winter wheat *per se*, as the shorter subsequent growing season requires spring wheat. It is also very likely that the temperature and conditions below the snow mean that the seed is dormant. Vernalization is an active process, so despite experiencing the required temperatures for vernalization, it would most likely not be met in these conditions. Developing wheat adapted to this farming approach is an area of opportunity for cereal breeders.

The separation of winter hardiness and vernalization is possible, as whilst winter wheat can have good frost tolerance and survive down to −20 °C, this is not true for all winter wheats. Therefore, whilst the vernalization response and winter hardiness have some genetic association, the two responses can be at least partially separated and so potentially independently selected (Galiba *et al*., 2009; Dhillon *et al*., 2010). The common link between the responses is the timing and level of expression of *VRN1* (Dhillon *et al*., 2010; Oliver *et al*., 2013). Other genes mapped as important in winter hardiness include *C-REPEAT BINDING FACTOR* (*CBF*) genes, which have undergone extensive copy number expansions in many cold hardy plants, including winter wheat (Pearce *et al*., 2013; Zhu *et al*., 2014; Babben *et al*., 2018). In wheat, *CBF-12*, *-14*, and *-15* are particularly associated with winter and frost hardiness, and located at the *Fr-A2* locus approximately 30 cM away from *VRN-A1* on chromosome 5A (Zhu *et al*., 2014). Copy number variation of *VRN1* and *CBF14* has been identified as a major determinant of frost tolerance, though this is dependent on the specific haplotype of the *Fr-A2* locus (Zhu *et al*., 2014; Sieber *et al*., 2016; Chen *et al*., 2024). Higher copy numbers of *CBF-14* and *VRN-A1* are associated with improved frost tolerance (Sieber *et al*., 2016; Chen *et al*., 2024). However, for *VRN-A1* this is likely because a higher copy number is associated with lower expression of *VRN-A1*, as there is a negative association of *VRN1* and frost tolerance (Diaz *et al*., 2012). This is potentially due to regulation of *CBF* genes by *VRN1*, as *CBF* and subsequent *COLD-REGULATED* (*COR*) genes show lower expression when *VRN1* is expressed (Kobayashi *et al*., 2005; Dhillon *et al*., 2010). There is some indication that this may be direct regulation, as VRN1 binds to the promoter of some *CBF* genes in barley (Deng *et al*., 2015).

There is also suggestion that the vernalization repressor *VRN2* may be involved in regulating frost tolerance, independent of *VRN1.* Despite its being a cereal-specific gene, *VRN2* overexpression in Arabidopsis was shown to increase frost tolerance (Diallo *et al*., 2010). Higher expression of the native *AtCBF* genes was shown in these transgenics, indicating VRN2 may also be involved in regulation of *CBF* expression (Diallo *et al*., 2010). In addition, one of the *VRN2* genes, *ZCCT2*, has been associated with the redox control of frost tolerance (Gulyás *et al*., 2014).

### Winter wheat and early plant architecture

The longer growing season that is facilitated by winter growth habit is caused by the delay in vegetative to reproductive development and therefore the increase in phyllochron, normally reflected as more leaves as well as an increase in the number of tillers that emerge (Fig. 1). The tiller emergence reflects the outgrowth of tiller meristems at the base of each node. The outgrowth of a tiller does not mean that these tillers will be converted to grain bearing stems, with many of the winter tillers being aborted later. Additionally, early winter tiller emergence occurs whilst the plant is vegetative. This often means that developmentally these tillers can appear more prostrate, which provides the winter crop with an increased potential to enable ground cover and so potentially facilitates its generation of a microclimate close to the soil that has a slightly higher temperature.

Notably, often agricultural practice drills winter wheat reasonably deep, and so the vegetative meristem is well protected from surface level frost until stem elongation starts. Generally, the increase in leaf and tiller growth during the vegetative period leads to an overall increase in plant biomass, tiller, and spikelet number that are ultimately very important factors in contributing to the higher yield observed with winter wheat. Here the interaction of VRN1 with other MADS-box genes has been identified to be important in regulating spike architecture and branching (Li *et al*., 2021). VRN1 is also involved in regulating plant height in both tetraploid and hexaploid wheat (Chen and Dubcovsky, 2012; Li *et al*., 2019; Liu *et al*., 2025). In hexaploid wheat a triple mutant of *vrn1* on the A, B, and D genomes flowered much later and was significantly shorter than the control genotype of Fielder (Liu *et al*., 2025). It would be interesting to understand if this regulation is maintained in a winter cultivar. Furthermore, interaction of VRN1 with the regulatory regions of *TaGA13ox1* and *TaGA20ox2* strongly supported the hypothesis that height regulation was via the gibberellin biosynthesis pathway (Liu *et al*., 2025).

Finally, from an agricultural perspective winter wheat has a longer period to establish than spring-drilled wheat. This is important under changing climate conditions as it enables a wider and deeper root system and so a greater ability to acquire nutrients and water during stressful periods. The root phenotype is influenced by winter habit genotypes. Two of the major seasonal habit genes have been linked with root traits: *VRN-B1* (*Vrn-B1*) regulates root biomass (Voss-Fels *et al*., 2018), and the insensitive *PPD-D1* (*Ppd-D1a*) allele has been associated with narrower and deeper roots compared with other *PPD-1* allelic types (Makhoul *et al*., 2024). This increased establishment compared with spring lines often means that a winter genotype is able to survive early spring drought. It also indicates that the winter genotypes may sequester more carbon through the extended root systems compared with spring genotypes.

## Opportunities for developing winter wheat

### Understanding *VRN1* as a target

Changing autumn and winter temperatures and durations, along with the variation in the rate and temperatures experienced with the onset of spring, means that there is a need and an opportunity to target the adaptation of cereal crops to enable the development of regionally robust cultivars. It is essential that incomplete vernalization and devernalization is avoided; however, simply removing the vernalization requirement would currently, in many environments, necessitate the transfer to a spring cereal cropping system that would lead to a significant reduction in yield and therefore increase in land area required.

Winter wheat is selected for in many breeding programmes via MAS. Here the established method of ensuring winter growth habit is to select for winter alleles of *VRN1* on the A sub-genome (Yan *et al*., 2003; Milec *et al*., 2023). The intron deletion has been shown to remove a regulatory element that is key in repressing *VRN-A1* expression (Fig. 2A). Therefore, this spring allele, like the promoter deletion, shows constitutively higher expression of *VRN1*. However, very counterintuitively, there is a report that an increase in copy number of the winter allele *VRN-A1* leads to an increase in vernalization requirement (Diaz *et al*., 2012). This is counterintuitive as an increase in copy number would suggest the potential for higher expression, similar to that of the gain of function allele that causes a spring growth habit. The copy number of the *VRN-A1* gene has been much harder to characterize due to the lack of sequence divergence between the copies. Determining copy number has relied on a SNP in exon 4 that can vary in *VRN-A1* copies and then TaqMan or KASP assays using relative intensity of fluorescence to determine copy number (Diaz *et al*., 2012). More recently, copy number has been determined by read depth comparison between genotypes confirming variation in copy number (Dixon *et al*., 2019). As the number of sequenced genomes increases, there is strong indication that copy number *per se* is not the sole contributing factor to determining vernalization duration as exceptions to the rule exist and it varies in spring habit lines with limited impact on flowering date (Jiao *et al*., 2025). Understanding the allelic types of *VRN-A1* is of upmost importance for generating targeted growth habit types. Additionally, understanding the contributions of the *VRN-B1* and -*D1* genes is also essential (Fig. 2). Similar intron deletions in either of these genes leads to facultative wheat, and this provides the current genetic definition of facultative wheat. However, this is not the only route to generate facultative growth habit wheat and so undoubtably needs revisiting. Equally exceptions exist for higher copy number of winter *VRN-A1* leading to a stronger vernalization response (Jiao *et al*., 2025) (Fig. 2). Therefore, considering the *VRN1* gene alone, we are unable to fully explain the alleles and the subsequent impact on vernalization and flowering.

### Beyond *VRN1* as a genetic target

**Fig. 3.**
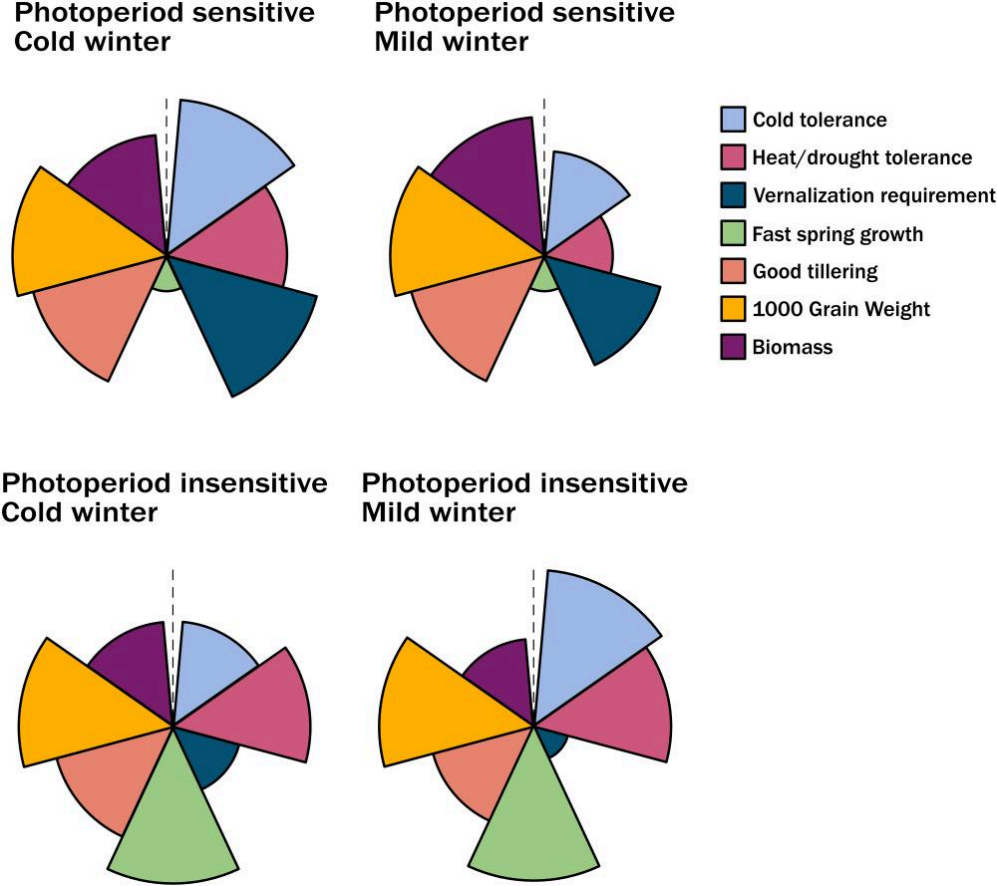
Proposed considerations for developing adapted wheat crops that account for multiple phenological characteristics. Through considering the strength of the type of winter growth required and possible novel allelic combinations to generate these, additional phenological traits can be included in crop development. These traits include the rate of plant establishment or the rate of development post-vernalization.

The vernalization potential of an environment will vary between regions, providing an opportunity to develop cereals where the vernalization response is mediated through alternative, non-vernalization-related genes. This would support flexibility for scenarios where the winter temperatures are highly variable. Here the recruitment of minor and major allele combinations of floral regulators needs to be considered to generate the growth rate required for specific cultivation conditions. This would represent a novel approach to the challenge of vernalization and enable the development of winter-grown cultivars that are tailored to the cultivation and environmental needs of a region (Fig. 3).

Delaying flowering through other genetic routes could include recruiting floral repressors in the *FLOWERING LOCUS T* or MADS-box clades (Hemming *et al*., 2012; Pieper *et al*., 2021). In central Europe, the requirement for reproductive development is likely to remain with the absolute need for vernalization, to prevent autumn development, but could be adapted to give cultivars that have a shorter duration of the vernalization period than is currently used. This would enable a vegetative winter development but with greater certainty that reproductive growth will happen in the spring. This will require understanding in more detail the alleles that determine the vernalization temperature and those that regulate the vernalization duration (Fig. 3).

In other regions, a different approach is needed and the requirement for a short-season winter wheat is established. Here vernalization is beneficial as it encourages vegetative growth and so soil cover over winter, reducing soil erosion in areas that are highly susceptible, for example the Great Plains of the USA. It also enables the establishment of biomass that can be grazed by livestock. Then rapid post-winter growth is required to enable rapid flowering to avoid or minimize the impact of drought conditions (Munaro *et al*., 2020). Therefore, vernalization requirement needs to be completely satisfied by the start of favourable growth conditions with no possibility of devernalization. Furthermore, it would be advantageous to combine such a requirement with alleles that promote early season growth. One strong candidate for this is the *FLOWERING LOCUS T3* (*FT3*) gene that is expressed under short days and lower ambient temperatures (Casao *et al*., 2011; Zikhali *et al*., 2017). *FT3* is a strong floral promoter and enables rapid floral meristem development in these early season conditions (Gauley *et al*., 2025). It has been inadvertently selected against in northern Europe where a slower apex development towards the end of winter and early spring is favoured to enable higher final yield potential (Gauley *et al*., 2025). However, in environments that require rapid post-winter growth to avoid end of season heat and drought, alternative alleles are enriched (Casao *et al*., 2011; Dreisigacker *et al*., 2021; Cheng *et al*., 2024). Targeting the selection and deployment of *FT3* alleles may offer a route to combine early apex growth with mild vernalization requirements (Fig. 3).

As described in the section ‘Winter wheat and cold hardiness’, one of the major benefits of vernalization is that it is closely linked with increasing a plant’s frost tolerance. Frost is a common occurrence in high latitudes and can cause a complete loss of crop. In more recent years the issue of frost damage has been increasing as the winter snow cover, which previously would provide a protective blanket, is diminishing. Therefore, generating winter hardy cultivars that are not dependent on vernalization but still remain vegetative is a major target for increasing the robustness of cereal yields. Once again, such a combination could be generated through targeted genetic selection of vernalization and flowering-related genes, including *VRN2*, *CBF*, *PPD-1*, *FT* genes, and MADS-box genes.

In lower latitudes the impact of frost is experienced later in plant development. In Australia the developing spike at the booting stage (GS45) and ear emergence (GS51) is vulnerable to frost damage, with the latter being more vulnerable due to no protection from the flag leaf (Zheng *et al*., 2015) (Fig. 3). Frost damage experienced during this later stage of development can be completely devastating through damage to the floral structures, developing grains, and supporting stems, and there is no developmental opportunity for the plant to recover (Barlow *et al*., 2015; Frederiks *et al*., 2015). It is probable that the vernalization genes are not impacting the response as late as the booting stage, but this has not yet been validated. However, through understanding the genes regulating frost response the re-expression of these genes could potentially be targeted to later in plant development, providing enhanced tolerance to frost. Models suggest that higher temperatures are accelerating the rate and timing of key developmental stages in wheat in both Australia and Argentina, particularly when sown earlier (Sadras and Monzon, 2006). Adjusting the sowing time is a method used to avoid frost damage; however, this comes with the risk of exposure to heat or drought damage if delayed too much (Ferrante *et al*., 2021). Increasing temperatures indicate the sowing window in Australia will have to shift earlier by up to 2 months to minimize the risk of frost damage, with a reduction in overall length of the growing season by up to 42 d (Zheng *et al*., 2012). Up to a week of this is predicted to occur during vegetative growth, which will likely impact vernalization requirement and potentially the yield potential (Zheng *et al*., 2012). The vernalization requirement for the lower latitude material could potentially be reduced or the rate of stem elongation also targeted to enable the timing of emergence to miss the frost windows. Although winter-habit cereals exhibit significant freezing tolerance during vegetative stages, they are highly susceptible to damage at comparatively moderate temperatures during reproductive stages. The later strategy inherently carries more risk as climate change is causing the shifting of this window.

Equally, the changing nature of the spring environment may also have a major impact on the vernalization response. There are a limited number of studies that address what the impact of slightly warmer springs will be on crop growth and the robust establishment of the vernalized state (Fradgley *et al*., 2023). A cut-off temperature will exist above which the plant will slowly devernalize. Breeding for a removal of this response, or for an increase in the devernalization temperature trigger would provide greater yield robustness. This particular response will be of increasing importance in areas that are experiencing earlier onset of warmer springs, including central and southern France and Spain. Again, maintaining the use of winter wheat as long as possible in these environments would support higher yield stability (Fig. 3).

An additional breeding target where vernalization can contribute is the increasingly prevalent dual-purpose crops, which are grazed and then harvested (as described above) with two crops per season (Fieser *et al*., 2006). Here, where cereals are currently poorly adapted, the higher profit crops are legumes, and a cereal crop would need to fit strictly to a growth period. Being able to target the vernalization response would enable higher yielding crops under short growth seasons.

One of the remaining major challenges of developing winter cereals is the time taken for generations. Under artificial conditions vernalization is completed in growth chambers between 4 and 6 °C under a low-light intensity short-day photoperiod (Cha *et al*., 2022). Plants are maintained in these conditions for 6–9 weeks depending on genotype and then transferred to standard glasshouse conditions of 16 h light: 8 h dark, 22 °C to grow to maturity. The average life-cycle of winter wheat is therefore 6 months (Cha *et al*., 2022). This limits the rate of genetic gain and places a significant time pressure on working with the crop as only two crossing seasons can be achieved per year to maintain the glasshouse cycles of the breeding companies. Therefore, a method to accelerate vernalization would enable faster development of important breeding lines. However, such a method should not alter the adaptive response of vernalization itself, for all of the reasons discussed above. The development of a speed vernalization protocol has been an advancement in this direction (Cha *et al*., 2022). Here it was identified that through early exposure of the seed to light at germination and then growth under low ambient temperature (10 °C) and extended long-day (22 h light: 2 h dark) photoperiod that the vernalization response could be significantly reduced in many winter wheat cultivars. Therefore, this reduced the breeding cycle for these cultivars without impacting the actual vernalization requirement. The same response was observed in barley. The expression of genes related to the vernalization pathway does not seem to be involved in the response, which suggests that this is different from vernalization and therefore could potentially be selected independently. To enable this, further characterization is needed to identify the genetic basis of the response and the impact that selecting for an alternative flowering-related pathway might have on crop development.

## Conclusion

Vernalization is an effective method of preventing premature floral development and enabling the autumn planting of wheat to maximize its cultivation. However, with changes in climate we are now presented with additional considerations to enable optimal adaptation of wheat over the winter season. Here we have reviewed our current understanding of vernalization and the possibilities to optimize this response as well as considering how plant development can be regulated over the winter season independent of the vernalization response. Through considering seed dormancy, plant establishment, tiller formation, rapid spring growth, and cold hardiness, we think these can be combined with the known alleles of *VRN1* and *PPD1* to enable climate robust wheat cultivars. This will require the generation and characterization of unusual allelic combinations that can be accelerated through the implementation of CRISPR technologies. Greater focus on identifying how specific traits are regulated beyond the impact of *VRN1* is important in developing new robust winter varieties. Through expanding our understanding of the evolutionary history of cereals and then utilizing this we anticipate that novel genetic targets will provide additional routes for seasonal adaptation beyond those recruited through domestication. Therefore, by combining our current molecular genetic knowledge with targeted understanding of additional genetic factors, we believe that a new suite of winter adapted cereals could be developed that are targeted to geographically specific winter environments. Importantly, phenological characteristics beyond that of seasonal winter development need to be considered to enable the rapid and useful adaptation of cereals.
